# Structural and Functional Characterization of Four Novel Fibrinogen Mutations in *FGB* Causing Congenital Fibrinogen Disorder

**DOI:** 10.3390/ijms23020721

**Published:** 2022-01-10

**Authors:** Eliška Ceznerová, Jiřina Kaufmanová, Žofie Sovová, Jana Štikarová, Jan Loužil, Roman Kotlín, Jiří Suttnar

**Affiliations:** 1 Department of Biochemistry, Institute of Hematology and Blood Transfusion, U Nemocnice 2094/1, 12800 Prague, Czech Republic; eliska.ceznerova@uhkt.cz (E.C.); zofie.sovova@uhkt.cz (Ž.S.); jana.stikarova@uhkt.cz (J.Š.); jan.louzil@uhkt.cz (J.L.); jiri.suttnar@uhkt.cz (J.S.); 2 Department of Biochemistry and Microbiology, University of Chemistry and Technology Prague, Technická 5, 16628 Prague, Czech Republic; jirina.kaufmanova@vscht.cz

**Keywords:** congenital fibrinogen disorder, *FGB*, hypofibrinogenemia, afibrinogenemia, dysfibrinogenemia, functional assays, scanning electron microscopy, homology modeling

## Abstract

Congenital fibrinogen disorders are caused by mutations in genes coding for fibrinogen and may lead to various clinical phenotypes. Here, we present a functional and structural analysis of 4 novel variants located in the *FGB* gene coding for fibrinogen Bβ chain-heterozygous missense BβY416C and BβA68S, homozygous nonsense BβY345*, and heterozygous nonsense BβW403* mutations. The cases were identified by coagulation screening tests and further investigated by various methods. Fibrin polymerization had abnormal development with decreased maximal absorbance in all patients. Plasmin-induced fibrin degradation revealed different lytic phases of BβY416C and BβW403* than those of the control. Fibrinopeptide cleavage measured by reverse phase high pressure liquid chromatography of BβA68S showed impaired release of fibrinopeptide B. Morphological properties, studied through scanning electron microscopy, differed significantly in the fiber thickness of BβY416C, BβA68S, and BβW403*, and in the fiber density of BβY416C and BβW403*. Finally, homology modeling of BβA68S showed that mutation caused negligible alternations in the protein structure. In conclusion, all mutations altered the correct fibrinogen function or structure that led to congenital fibrinogen disorders.

## 1. Introduction

Coagulation factor I, fibrinogen, is a hemostasis protein, with its primary role being to strengthen blood clots. Fibrinogen’s unique properties are based on its six-chain dimer structure that is formed by three pairs of polypeptide chains (Aα, Bβ, and γ). Each polypeptide is coded by its own gene (*FGA*, *FGB,* and *FGG*). Newly synthesized chains are assembled into three-chain monomers (Aα-Bβ-γ) and, subsequently, into the whole molecule, which is secreted out of hepatocytes [1,2,3].

A mutation in one of the genes may cause a congenital fibrinogen disorder (CFD). These ailments can be classified into four different groups based on the levels of functional and total fibrinogen in plasma. The first two groups—afibrinogenemia and hypofibrinogenemia—present with a lower level of both parameters. Therefore, these disorders lead to quantitative fibrinogen deficiencies. The third group—hypodysfibrinogenemia—reflects the decreased level in total fibrinogen, and, moreover, fibrinogen molecules have an impaired function. The last group—dysfibrinogenemia—shows normal levels of total fibrinogen, but the functional activity may be impaired by the mutation causing qualitative fibrinogen deficiencies [4].

The severity of clinical manifestation of CFDs is highly heterogeneous, from asymptomatic to bleeding and/or thrombotic, and is mainly dependent on fibrinogen levels [5]. In quantitative diseases, patients with afibrinogenemia frequently have a bleeding phenotype; however, cases of thrombosis have been reported as well. Patients with hypofibrinogenemia are mostly asymptomatic [6]. In qualitative diseases, mutant fibrinogens in the heterozygous state circulate in the blood as homo- or heterodimers. The compositions of these molecules affect the function of fibrinogen and, therefore, its clinical phenotype [5].

Abnormalities in the *FGB* gene are less common compared to the *FGA* and *FGG* genes. Until 2016, 1215 molecular abnormalities were individualized in the GEHT database. Of these, 626 were Aα mutations, 154 Bβ mutations, and 435 γ mutations. The most recent update in June 2020 released 50 new variants only in *FGB* and *FGG* [7]. Bβ mutations are mostly caused by missense or late-truncating nonsense mutations in codons for coding the C-termini of the Bβ chain [8]. Interestingly, it is mainly the structure of the Bβ chain that is a rate-limiting factor for fibrinogen expression, particularly the individual residues and a correctly folded three-dimensional structure of the Bβ chain C-terminus and its incorporation into the assembling protein [9]. Nevertheless, although the number of studied mutations is quite substantial, it still remains unclear how some amino acids are involved in fibrinogen assembly, structure, and function. Therefore, the identification and characterization of new congenital fibrinogen variants are necessary for a better understanding of fibrinogen synthesis and function.

Here, we identified four novel mutations located in the *FGB* gene—heterozygous missense BβY416C and BβA68S, homozygous nonsense BβY345*, and heterozygous nonsense BβW403* mutation. We further investigated these mutations through turbidimetric analysis, fibrinopeptides (Fps) release measurement, scanning electron microscopy (SEM), and molecular modeling. Our results provide a correlation between the functional and/or structural modifications of these novel mutations and the patient’s clinical phenotype.

## 2. Results

Patients were included in this study on the merit of their routine coagulation results indicating CFD (Table 1). Subsequent genetic analysis revealed that each of them had a novel mutation in the *FGB* gene. Therefore, we used specialized biochemical and bioinformatical methods to characterize their fibrinogen properties, except for P3, who bore a homozygous mutation and had undetectable levels of fibrinogen in his plasma.

### 2.1. Coagulation Screening Tests of Patients with CFD Suspicion and Genetic Analysis

Patient 1 (P1) was a 79-year-old man diagnosed with vitamin B12 anemia with no bleeding or thrombotic history. He presented with decreased levels of functional and plasma fibrinogen, and with prolonged thrombin (TT) and reptilase time (RT) (Table 1). We examined his fibrinogen exons for CFD and found a novel heterozygous missense mutation—fibrinogen Karlovy Vary—in position c.1337A>G in exon 8 of the *FGB* gene. This change led to substitution p.Y416C (mature chain missing 30 amino acids of signal peptide).

Patient 2 (P2) was a 17-year-old female with a lower level of functional fibrinogen and prolonged TT (Table 1). She suffered from repeating epistaxis twice per week after otitis in 2016. Therefore, she was treated with Ascorutin but with no effect. She also had mild post-traumatic cutaneous bleeding. Initial genetic sequencing revealed a novel heterozygous missense mutation in position c.292G>T in exon 2 of the *FGB* gene, p.A68S. We designated it as fibrinogen Svetec. Her 44-year-old mother (M2) had similar routine clotting time tests results (Table 1) with no bleeding history. Her 26-year-old brother (B2) had normal values of coagulation screening; however, he reported mild umbilical bleeding and repeating epistaxis treated successfully with Gelaspon. Interestingly, the genetic analysis found the same mutation in M2 *FGB* exon 2 as in the proband, but not in B2.

Patient 3 (P3) was an asymptomatic newborn with pathological coagulation test results (Table 1). All of the values were prolonged, and the fibrinogen levels were null. DNA sequencing identified a novel homozygous nonsense mutation in exon 7 of the *FGB* gene c.1125C>A, causing the formation of premature codon p.Y345*. This mutation was designated as fibrinogen Trinec. His mother had normal clotting times, whereas his father (F3) presented with shortened RT (Table 1). Unfortunately, the genetic analysis of P3’s parents was not possible due to the unavailability of their DNA.

Patient 4 (P4) was 47-year-old woman with slightly decreased levels of both functional and plasmatic fibrinogen with prolonged TT and shortened RT (Table 1). She was diagnosed with antiphospholipid syndrome (APS), suffered from pulmonary embolism in 2015, and had three miscarriages during eight weeks of pregnancy. She is treated with the warfarin (12 mg/day). DNA analyses revealed a novel heterozygous nonsense mutation c.1298G>A in exon 8 of the *FGB* gene, p.W403*. According to the place of patient residence, we named this fibrinogen as fibrinogen Most.

### 2.2. Characterization of Each Patient’s Fibrinogen Behavior

**Figure 1:**
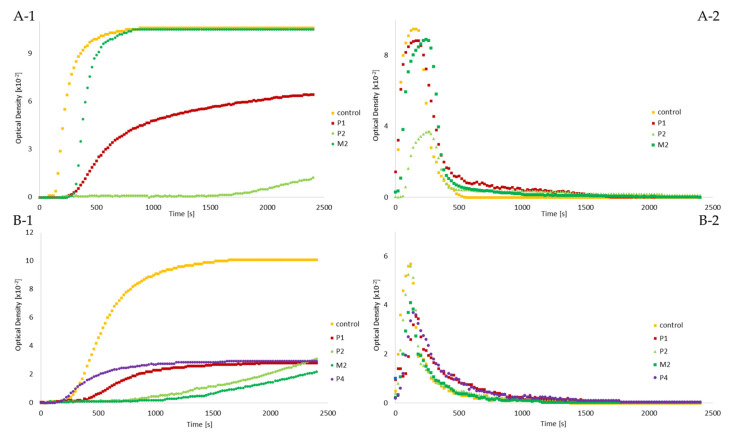
Fibrin polymerization (1) and plasmin degradation (2) analysis of patients compared to control revealed differences; (**A-1**,**A-2**) plasma samples’ measurement; (**B-1**,**B-2**) purified fibrinogen samples’ measurement; Abbreviations: P1: patient 1; P2: patient 2; M2: mother of patient 2; B2: brother of patient 2; P4: patient 4.

Thrombin-catalyzed polymerization and further plasmin degradation revealed differences between the patients and control samples (Table 2 and Figure 1).

In P1, plasma fibrin polymerization, maxOD (1.6×), and slope (4.3×) were decreased. Lag time (2.4×), T90% (3.9×), T50% (1.3×), and T100% (3.0×) were prolonged compared to the control sample. Purified fibrin polymerization had a similar course as the plasma sample. MaxOD (3.6×) and slope (4.1×) were diminished, and lag time (1.7×), T90% (1.2×), and T50% (1.8×) were also prolonged. Only T100% (1.1) fibrinolysis was comparable to control (Table 2).

P2 had severely prolonged lag times, 13.9× for plasma, and 3.6× for purified fibrinogen. Therefore, T90% was also extended (5.6× plasma, 2.3× purified fibrinogen). In plasma, the maxOD was 8.2× decreased and slope 24×. In the purified fibrinogen sample, 3.2× maxOD and 4.8× slope were noted. The fibrinolysis was slightly prolonged, T50% 1.5× for plasma, and 1.1× for purified fibrinogen. T100% was undetectable in plasma because the lytic curve did not reach 0.03 OD, and for purified fibrinogen was comparable with control (1.0×).

M2 had comparable fibrin development kinetics of the plasma sample as the control, except the 2.1× prolonged lag phase. Plasmatic fibrinolytic parameter T50% was 1.4× prolonged, as well as parameter T100% (3.4×). However, the purified fibrinogen polymerization and fibrinolysis curve behaved differently. Lag time (5×) and T90% (2.3×) were prolonged, whereas the maxOD (4.6×) and slope (6.9×) were decreased. T50% was comparable to control (1.1×), and total fibrin degradation was slightly faster (1.2×).

The purified fibrinogen analysis of P4 revealed a comparable lag time (1.1) and T90% (0.9), 3.4× decreased maxOD and slope, 1.9 × T50%, and 1.3 prolonged T100% (Table 2). Unfortunately, P4’s plasma turbidity analyses were not measured due to the unavailability of the patient’s plasma.

### 2.3. Quantification of Thrombin-Catalyzed Cleavage of Fps

**Figure 2:**
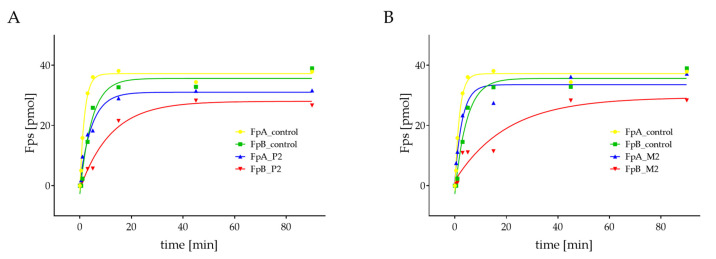
Measurement of patients’ Fps (blue and red) compared to controls (yellow and green). (**A**) P2 had prolonged cleavage and a decreased amount of FpB as well as M2 (**B**); Abbreviations: P2: patient 2; M2: mother of patient 2.

The amount of released Fps was measured in the patients with missense mutations BβA68S—P2 and M2 by reverse phase high liquid pressure chromatography (RP-HPLC) (Figure 2). P2 and M2 had prolonged cleavage and decreased amounts of fibrinopeptide B (FpB) compared with that of the control. Moreover, P2 also had a slower release of fibrinopeptide A (FpA), and their total concentration was lower compared to controls.

### 2.4. Morphologic Differences of Patient’S Fibrin Clots Compared to Controls

**Figure 3:**
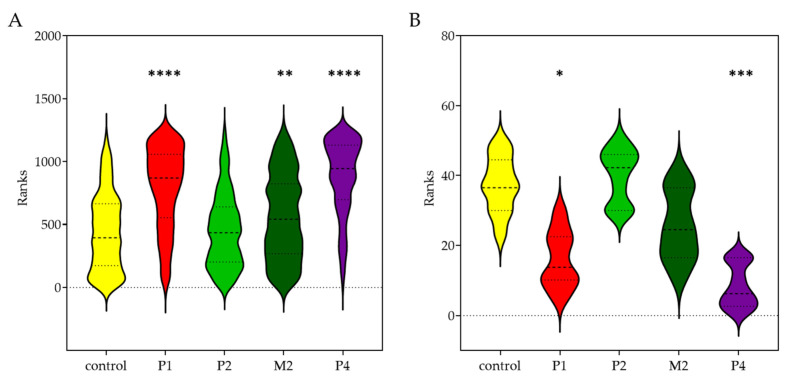
Violin plots represent the results of (**A**) fiber thickness measurement, where the patient samples were compared to control. The Kruskal–Wallis test revealed significant difference in P1 (****; control vs. P1, *p* < 0.0001), M2 (**; control vs. M2, *p* = 0.011), and P4 (****; control vs. P4, *p* < 0.0001). The fiber thickness of P2 was comparable to controls (control vs. P2, *p* > 0.9999). (**B**) average no. of fibers per 1µm^2^ measurement, where the patient samples were compared to control. The Kruskal–Wallis test revealed significant difference in P1 (*; control vs. P1, *p* = 0.0306) and P4 (***; control vs. P4, *p* = 0.0009). P2 (control vs. P2, *p* > 0.9999) and M2 (control vs. M2, *p* = 0.6654) had a comparable no. of fibers to controls. Abbreviations: P1: patient 1; P2: patient 2; M2: mother of patient 2; P4: patient 4.

**Figure 4:**
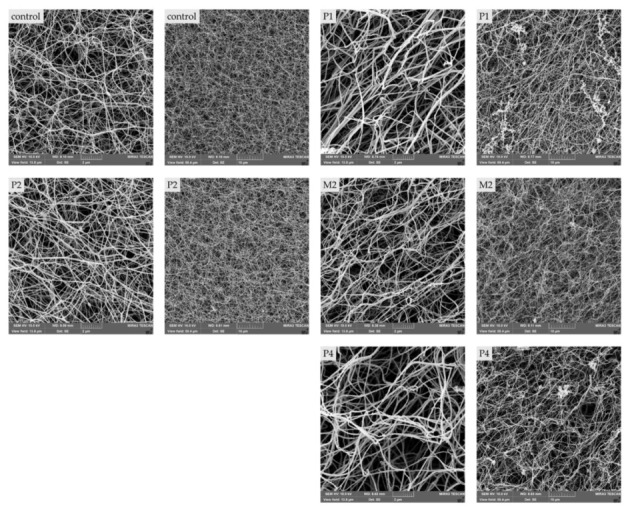
Representative SEM pictures of patients and control. Abbreviations: P1: patient 1; P2: patient 2; M2: mother of patient 2; P4: patient 4.

Clot structural properties measured by SEM were determined between control and patient samples (Table 3). P1, M2, and P4 had significantly thicker fibers as compared to the controls (Figure 3A); interestingly, P2 revealed no statistical difference in fiber thickness (Figure 4).

Furthermore, fiber density analyses showed a significantly lower number of fibers in P1 and P4 clots (Table 3) (Figure 3B). P2 and M2 had similar numbers of fibers.

### 2.5. Protein Modeling of BβA68S

**Figure 5:**
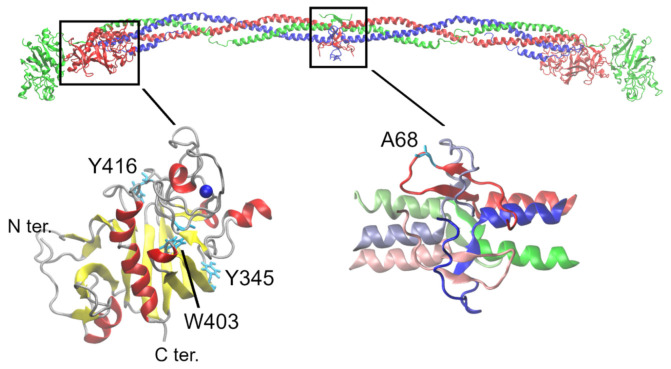
Position of the mutated amino acids within fibrinogen. Above: Black boxes overlaying the crystal structure 3GHG show regions where the detected mutations are situated. Aα chain is shown in blue, Bβ chain in red, and γ chain in green. Below left: fibrinogen-related domain of the Bb chain; three mutated amino acids are shown in cyan. Below right: adjusted part of the E-domain of fibrinogen that was the subject of homology modeling with shown amino acid BbA68. Chains of the second half-molecule are depicted in light colors.

Models of wild-type (WT), BβA68S fibrinogen, and crystal structure varied only in the regions where no template structure was supplied (Figure 5). The root-mean-square deviation (RMSD) between the backbone atoms of the models was 0.53 Å, indicating that they were almost identical. A comparison of RMSD values for the backbone atoms of the crystal structure with models of WT and mutant provided values of 0.58 and 0.60 Å, respectively. This finding suggested that modeling influenced the WT fibrinogen structure; therefore, the mutant model should be compared with the WT model, rather than with the crystal structure.

Next, we focused on the parts of the molecule that differ in the model structures-BβG61–C76 and Aα’S31–E39 (mature protein numbering). The RMSD of the backbone atoms of the WT and mutant models was within this region 1.92 Å, meaning that these two structures were different. RMSD calculations further confirmed that the mutant model was closer to the crystal structure (1.24 Å) than the model of the WT (2.15 Å). From this result, it might be hypothesized that the conformation of the mutant is more stable than that of the WT.

**Figure 6:**
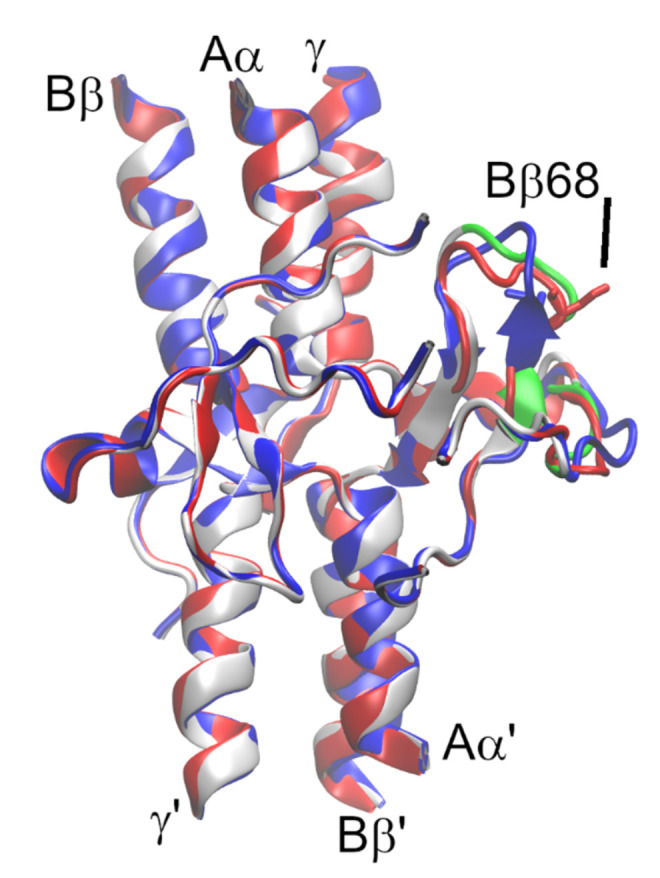
Fit of homology models of mutant (red) and WT structures (blue) onto crystal structure 3GHG (grey). Parts of the crystal structure missing in the alignment are shown in green.

To characterize the changes between structures in a qualitative manner, we compared the secondary structure among parts of the molecule affected by the mutation (Figure 6) (Table S1). To do so, we used the Kabsch–Sander classification of secondary structure that is based on hydrogen bond patterns within the protein. We indirectly described the altered hydrogen bonds’ architecture. In the N-terminus of the Bβ chain, the turn (amino acids BβD69–G73) was the dominant secondary structure element of the mutant model and in the crystal structure. The bend (amino acids BβD69–L72) dominated in the secondary structure of the WT model. Whereas the turn is defined by the presence of a hydrogen bond between i and i + n residue, no hydrogen bonds are necessary for the bend. In the bend, the angle between i − 2, i, and i + 2 residues must be larger than 70°. The N-termini of the Aα’chains of crystal models were mainly disordered loops and vary in the geometry of residues Aα’S37–D42, which form a turn (undefined type) in the mutant and a 3_10_-helix in the WT model of fibrinogen. The 3_10_-helix is defined by at least two consecutive β-turns, whereas the type of turn(s) are not defined for the mutant. We did not determine the hydrogen bond pattern of the mutant by its visualization in SwissPDBViewer due to the different definitions of hydrogen bonds in SwissPDBViewer and Procheck. The secondary structure in this region of the crystal structure was recognized as the turn.

In conclusion, structural modeling revealed only a negligible change caused by the altered hydrogen bond pattern that was induced by the fibrinogen structure due to the BβA68S mutation.

## 3. Discussion

### 3.1. Molecular Characteristic of 4 Novel Fibrinogen Variants

We detected a novel heterozygous mutation BβY416C (c.1337A>G) in P1 and designated it as fibrinogen Karlovy Vary. This missense mutation is located in exon 8 of the *FGB* gene, which is known as a frequent location for quantitative mutations. Exon 8 encodes for the highly conserved C-terminus of the Bβ chain [10]. Recombinant studies on transfected cells have demonstrated the importance of these globular structures for fibrinogen assembly and secretion [9].

Several missense mutations located in the surroundings of BβY416C were described earlier [7]. Fibrinogen India BL-267 and BL-377 (BβR415T) were found to be homozygous mutations causing afibrinogenemia, most likely by secretion inhibition [11]. As another example, fibrinogen Turkish (BβN413K) was studied by structural analysis, confirming that fibrinogen secretion is affected by changes in the Bβ chain C-terminus [12].

Fibrinogen Svetec is the first described case of a heterozygous missense mutation BβA68S in exon 2 of the *FGB* gene. We detected this mutation in P2, her mother (M2), but not in her brother (B2). BβA68 is located in sequence BβH67-P70, which forms a β turn in a highly conserved sequence.

Mutations in the same position BβA68 were described in six unrelated families but with substitution BβT68–fibrinogen Naples = Milano II, Shizuoka (Yonekawa et al., 16th Congress of International Society on Thrombosis and Haemostasis, Florence, 1997, PS-2550), Chinese, and Hamamatsu [13,14,15,16,17]. In all cases, a mutation led to dysfibrinogenemia and, in homozygous patients, caused thrombosis. Fibrinogen Šumperk BβH67L is an example of a mutation close to ours. Interestingly, this substitution causes hypofibrinogenemia. Molecular modeling showed the loss of a hydrogen bond between the BβH67 and BβD69, causing alternation in fibrinogen assembly and secretion [18]. Since both serine and threonine are polar, aliphatic amino acids, one would expect a similar manifestation of both BβA68S and BβA68T mutations.

The last two variants we revealed in the *FGB* gene were fibrinogen Trinec (BβY345*) and fibrinogen Most (BβW403*). Both mutations led to the formation of a premature codon. In the case of fibrinogen Trinec, it was caused by a homozygous mutation in exon 7 position c.1125C>A changing the tyrosine codon to a stop codon. In fibrinogen Most, a heterozygous change in c.1298G>A of exon 8 substituted tryptophan.

As in the case of fibrinogen Karlovy Vary, mutations were identified in the C-terminus of the Bβ chain, which is a highly conserved region among vertebrates. Kotlín et al. described variant heterozygous BβN351K (fibrinogen Rokycany) causing hypofibrinogenemia. It changes the conformation of γ turn BβG350–BβA352 and its neighborhood, an important part of the Bβ chain for correct fibrinogen folding [19]. Duga et al. studied the expression of BβL353R (afibrinogenemia Milano) and BβG400D (afibrinogenemia Milano II) in COS-1 cells. This experiment showed that fibrinogen molecules were synthesized and assembled intracellularly, but the truncated fibrinogens could not be detected in cell media. In both cases, mutations were homozygous and led to afibrinogenemia [20]. Casini et al. revealed a novel case of hypofibrinogenemia with protein modeling of heterozygous BβW403L (fibrinogen Swiss). The mutation caused changes of hydrogen bonds and amino acids in the neighborhood of BβL403 [12]. Hanss et al. reported the loss of 59 C-terminus amino acids in the case of heterozygous mutation BβW402* (fibrinogen Lyon II) that was not detected in plasma due to altered fibrinogen secretion [21]. The final example is heterozygous mutation BβC407*, fibrinogen St Kilda, where the authors assumed that the destabilization and loss of an intra-molecular disulfide bridge between BβC394 and BβC407 results in the intracellular degradation of fibrinogen. Thus, their patient was diagnosed with hypofibrinogenemia [22]. A study conducted by Zhang and Redman on Bβ chain domains that are involved in fibrinogen assembly and secretion showed that the C-terminus side of the amino acid is important for fibrinogen assembly and secretion [23].

We hypothesize that fibrinogens Karlovy Vary, Trinec, and Most are likely assembled. However, due to the mutation-induced changes, fibrinogens could not be secreted out of hepatocytes. Consequently, the levels of total and functional fibrinogen in the patient’s plasma are lower. Moreover, fibrinogen Trinec was undetectable. Therefore, these mutations—BβY416C and BβW403*—might well be the cause of P1’s and P4’s mild hypofibrinogenemia, and in the case of the homozygous BβY345* mutation, it is most likely the direct cause of the patient’s afibrinogenemia. Finally, fibrinogen Svetec is the novel missense mutation detected in BβA68, with a normal level of total fibrinogen and decreased level of functional fibrinogen. These results, in combination with the literature, indicate that mutation BβA68S is the most likely cause of dysfibrinogenemia in P2 and M2.

### 3.2. Correlation between Mutations, Fibrinogen Function, Clot Structures, and Clinical Phenotype

Since some patients suffered from bleeding or thrombotic episodes, we decided to investigate the functional and morphological properties of all novel fibrinogen variants of this study except for fibrinogen Trinec, as P3 had undetectable levels of plasma fibrinogens. Moreover, only in the quantification of Fps, samples of patients with missense mutation BβA68S were analyzed due to the unavailability of other patients’ plasma.

#### 3.2.1. Fibrinogen Karlovy Vary and Fibrinogen Most

In patients with mild hypofibrinogenemia and fibrinogens Karlovy Vary and Most, no functional abnormalities were observed during conversion from fibrinogen to fibrin, although there was decreased polymerization due to lower levels of plasmatic fibrinogen (Figure 1). Moreover, during fibrinolysis, the clot lytic phase was longer than those of the control (Figure 1).

The SEM images of clots showed significant differences in measured properties of hypofibrinogenemia patients and those of the control (Figure 3). The clot structures were composed of significantly thicker fibers that were sparsely packed together (Table 3). A similar fiber thickness was measured in two unrelated patients diagnosed with hypofibrinogenemia with heterozygous mutation BβT444* (Fibrinogen Martin IV). It was assumed that this abnormal structure was caused by lower levels of fibrinogen in patients’ plasma [24].

In hypofibrinogenemia, the mutated protein is not presented in circulation. Therefore, the morphological and functional abnormalities of our patients are not caused by fibrinogen mutations. Nevertheless, the clot formation and its properties depend on many variables, including the quantity and quality of fibrinogen, concentration of calcium, local pH, ionic strength, and thrombin concentration [25,26]. The final clot structure reflects the reaction between the thrombin and fibrinogen; therefore, their concentrations are the main determinant [27]. The actual thrombin concentration at the time of polymerization has a particularly important impact on clot structure. High concentrations produce thinner fibers packed more densely. In contrast, low thrombin concentrations (<0.1 U/mL) produce thick fibers sparsely packed together. These thick fibers also increase fibrin turbidity within the fibrin formation [28].

Fibrin degradation is affected by clot architecture. Plasmin generation by tissue plasminogen activator (t-PA) is slower in clots with thinner fibrin fibers than those with thicker fibers [29]. Moreover, clots formed with thinner fibers are more resistant to fibrinolysis because they are tightly packed and have more fibers to cleave [27].

Other studies have shown the differences between in vivo and in situ clot formation, where the thrombin is generated in the presence of cells and other plasmatic proteins. For example, the addition of calcium to the thrombin reaction shortens the start of clotting and forms thicker fibers than the reaction without the calcium [25]. Some plasmatic proteins, such as antithrombin, have a similar outcome as calcium concentration. The presence of antithrombin decreases the concentration of active thrombin and results in a prolonged lag phase and thicker fibrin fibers. The lag phase, in the presence of albumin, hemoglobin, and γ-globuline, is shortened due to their influence on macromolecular interactions [30].

Patients with hypofibrinogenemia are frequently asymptomatic, as was the case for our patient with fibrinogen Karlovy Vary. Nevertheless, cases of bleeding linked with trauma or surgery have been reported. The thrombotic phenotype is not frequent, but several cases have been described [6,7]. Moreover, women with hypofibrinogenemia are at a high risk of clinical manifestation, particularly during pregnancy [31]. The patient with fibrinogen Most suffered from thrombosis and three miscarriages. However, this patient was also diagnosed with APS, which is a systemic autoimmune disorder characterized by thrombosis and/or miscarriages [32]. Therefore, it is difficult to determine whether the fibrinogen Most contributed to the patient’s clinical phenotype, or if it was caused only by APS.

#### 3.2.2. Fibrinogen Svetec

Functional analysis of fibrinogen Svetec showed markedly prolonged polymerization, with a less steep slope of P2 in plasma as well as in purified fibrinogen samples. Interestingly, plasma polymerization of the proband mother (M2) was comparable to control, except for the prolonged lag phase. However, within the purified fibrinogen, polymerization differences in maximal absorbance were revealed (Figure 1). Furthermore, Fps release kinetics measurements revealed another functional defect of fibrinogen Svetec (Figure 2). FpA were released more slowly than those of the control, and fewer FpB were detected. During fibrin degradation, the only difference was in the slightly prolonged dissolution of P2’s plasma sample. The clot structure of P2 was comparable to those of the control with fiber thickness and density. In the M2, the clot architecture was only more densely packed (Figure 3).

Mullin et al. studied the behavior of recombinant fibrinogen Naples, BβA68T. They found that the polymerization lag phase was prolonged, with a lower slope, and the fibers thickness was comparable with the control in the case of normal thrombin concentrations [33]. Lord et al. found a two-fold slower rate of FpB release from BβA68T than control [34]. Koopman et al. proposed that mutation BβA68T decreases the release of FpA and FpB due to defective binding of thrombin [13]. Moreover, Mullin et al. suggested that mutation BβA68T impaired the enzymatic phase of polymerization since it alters the subtle conformational change in fibrinogen the E domain that occurs between the cleavage of FpA and FpB, and modulates the lateral aggregation of protofibrils [33].

Therefore, we hypothesize that the different polymerization of BβA68S is most likely due to a delay in FpB cleavage and impaired lateral aggregation. This hypothesis is supported by the fact that the patients had prolonged TT and RT, prolonged and decreased polymerization, and impaired kinetics of Fps release.

The clinical phenotype of fibrinogen Naples (BβA68T) is associated with thrombosis in the homozygous state due to defective thrombin binding and its increased concentration in the circulation [13]. Patients in a heterozygous state were reported asymptomatic (Yonekawa et al., 16th Congress of International Society on Thrombosis and Haemostasis, Florence, 1997, PS-2550) [35].

Our patient with mutation BβA68S (P2) manifested bleeding symptoms, and her mother (M2) was asymptomatic. Interestingly, her brother had a mild bleeding phenotype without the BβA68S mutation being detected. Functional fibrinogen changes could also be caused by post-translational modifications. Brennan et al. published mutation BβG401V, which was presented in one carrier as hypodysfibrinogenemia and in others as hypofibrinogenemia. They found post-translational modifications of circulating fibrinogen causing a higher proportion of disialo-isoforms in the carrier with hypodysfibrinogenemia that causes the functional defect of fibrinogen [36]. Therefore, this finding suggests that the bleeding tendency of both siblings may not be associated with the mutated fibrinogen.

It is also known that fibrinogen is vulnerable to oxidative stress more than other plasmatic proteins. Fibrinogen alters fibrin polymerization, clot structure, and its susceptibility to fibrinolysis [37]. The limitation of this study is the lack of plasma samples to explore more detailed correlations between the bleeding phenotype of P2 and B2 and their fibrinogen.

### 3.3. Homology Model of Fibrinogen Svetec

Since the missense mutation BβA68S causes changes that influence the function of fibrinogen Svetec, we investigated this substation by homology modeling. Our model showed only minor changes in the structure of BβG61—C76 and Aα’S31—E39 (mature protein numbering).

The homology model of the BβA68S mutant was compared with the WT model, not with the template structure of the 3GHG crystal. This approach was performed to analyze the structures obtained by the same method that were influenced by the same systematic error. We used an identical template structure, defined the BβC65-Aα’C36 disulfide bridge, and removed parts of the crystal structure in the spatial vicinity of the mutation because we wanted to study the impact of the mutation on its surroundings.

Modeller constructs models in the way that first places the amino acids with the assigned alignment on the position of the template amino acid. It then adds amino acids corresponding to those missing in the alignment so that they fulfill the stereochemical demands for amino acids within proteins. The entire model consequently undergoes geometry optimization. By removing amino acids from the template, we enabled these amino acids to be placed in stereochemically more convenient positions than those in the crystal. This strategy is necessary due to the different properties of alanine and serine.

When interpreting the results of theoretical methods such as homology modeling, one must be aware that specific results are always obtained, irrespective of their quality. Hence, more models are computed, and the best of them is chosen. Note that models reflect the state corresponding to the static state of protein, such as in the crystal. In reality, proteins are subject to internal motion, and they interact with solvent. None of these processes is reflected by models. Therefore, the stability of loose secondary structure elements, such as turn(s) obtained by homology modeling, is questionable, as is the conformation of disordered parts of the molecule. In vivo, chaperones participate in protein folding by overcoming energetic barriers. Therefore, theoretical models are not able to capture the influence of chaperons.

Taken together, our model of the BβA68S mutation showed only minor alternations in the secondary structure of both Bβ as well as the surrounding part of the Aα’ chain of fibrinogen. The stability of these hydrogen bonds in solution is questionable and cannot be judged from the presented data. Therefore, the interpretation of these models must be taken very carefully, and we agree with Koopman et al., [13] that the mutation of BβA68 to polar amino acids influences the interaction of fibrinogen with thrombin, and that dysfibrinogenemia does not originate from the structural properties of fibrinogen but from its functional properties.

## 4. Materials and Methods

All reagents used were of analytical grade and obtained from Merck (Darmstadt, Germany), apart from the primers (Generi-Biotech, Hradec Kralove, Czech Republic).

### 4.1. Blood Collection and Coagulation Screening Tests

Investigated patients and healthy volunteers agreed to this study. Blood samples for biochemical and genetic analyses were collected with informed consent. The study was approved by the Institute of Hematology and Blood Transfusion Ethics Committee, and all samples were obtained in accordance with the regulations of the ethical committee of the institute and with the Declaration of Helsinki.

Citrated blood samples of both, patients and controls, were collected into plastic tubes filled with 1 mL of 3.8% trisodium citrate by venipuncture to a 9 mL final volume. Platelet-poor plasma was prepared by centrifugation at 1400× *g* at room temperature (RT) for 10 min. Samples were transferred into Eppendorf tubes and stored at −80 °C until use. Concentrations of fibrinogen in plasma samples were adjusted to 1 g/L for all analyses.

Routine coagulation tests were measured on a STAR-R coagulation analyzer (Diagnostica Stago, Asnieres-sur-Seine, France). Plasma levels of functional fibrinogen were determined by a thrombin time method (Clauss method) [38], and the total level of fibrinogen by an immunoturbidimetric assay using anti-human fibrinogen goat antiserum, performed on a UV-2401PC spectrophotometer (Shimadzu, Kyoto, Japan) using the manufacturer’s kit (κ-ASSAY Fibrinogen; Kamiya Biochemical Company, Seattle, WA, USA).

### 4.2. Fibrinogen DNA Sequencing

Screening of all exons coding for fibrinogen was performed by amplification of patients DNA by PCR and, subsequently, sequenation by the Sanger method using a CEQ 8000 genetic analysis system (Beckman coulter Inc., Fullerton, CA, USA). Patient results were compared with DNA sequences of verified 100 healthy and unrelated controls measured by the same Sanger sequencing. Mutations were described according to Human Genome Variation Society guidelines [39], using both c.DNA descriptions based on NCBI reference sequence NM_005141.5 for *FGB* and protein descriptions based on the mature chain of NP_005132.2 (signal peptide contains 30 amino acids).

### 4.3. Fibrinogen Purification

Fibrinogen was purified by the precipitation of citrated plasma of patients and healthy controls according to the method of Brennan et al. [40]. Briefly, 22.5% ammonium sulfate was added to samples to precipitate at 4 °C for 30 min, and then centrifuged for 5 min and 4 °C at 5000 rpm. The pellet was dissolved by 25% ammonium sulfate in the original plasma volume and centrifuged using the same conditions as described previously. This step was repeated, and the precipitate was finally dissolved in the original plasma volume by adding TRIS buffer pH 7.4. Purified fibrinogens were subsequently used for further analysis.

### 4.4. Fibrinogen Polymerization and Fibrinolysis

The turbidity of fibrin polymerization was measured on both purified fibrinogen and plasma samples of all patients and healthy control. Briefly, samples were diluted with TRIS buffer pH 7.4 at a ratio of 1:3. The reaction was activated with 50 µL of thrombin (0.1 U/mL final concentration) with 2 µL CaCl2 (8 mM final concentration). Fibrinolysis was activated by the addition of thrombin (12 NIH U/mL final concentration), plasminogen (0.15 NIH U/mL, final concentration), t-PA (0.3 μg/mL final concentration), and CaCl_2_ (8 mM final concentration).

In both methods, the optical densities (OD) were detected at 350 nm every 20 s for 40 min using an ELISA reader Synergy HT (Bio-tek Instruments, Winooski, VT, USA). The lag time (time to 0.5 OD), maximal absorbance (maxOD), slope (maxOD/min), and T90% (time to 90% of maxOD) were calculated from polymerization curves for all. Fibrinolysis was analyzed by the time to reach 0.03 OD within the fibrin degradation T100% and by T50%, which is time from 50% of polymerization maxOD to 50% of fibrinolysis maxOD. The values were calculated using Microsoft Excel. Samples were measured in triplicate.

### 4.5. SEM

Plasma samples of volume 50 µL were clotted with thrombin (2 U/mL and 17 mM CaCl_2_ final concentration) in a microplate for 3 h at room temperature. The clots were fixed by 4% formaldehyde overnight, carefully washed with cacodylate buffer, and dehydrated with increasing ethanol concentrations (30%, 50%, 70%, 90%, and 2 × 100%). Samples were resuspended in acetone with the use of liquid CO_2_ in a critical point drier. Finally, clots were covered with a 10 nm layer of gold film and measured on a TESCAN MIRA 3 (Tescan Brno s.r.o., Brno, Czechia). Clots of each patient were made in duplicate and the fiber thicknesses and densities were evaluated using ImagineJ 1.33 data analysis software (National Institutes of Health, Bethesda, MD, USA). The fibers diameters were determined from 250 values (25 per image). The fiber density was analyzed by shotgun plot analysis.

### 4.6. Quantification of Fps Cleavage by RP-HPLC

Reactions were initiated with the addition of thrombin (final concentration 0.9 NIH U/mL) to 150 µL of diluted plasma samples (1:3 with TRIS buffer pH 7.4) with O-phenanthroline (final concentration 12 mM). For each sample, eight reactions proceeded in the time profile 0, 0.5, 1, 3, 5, 15, 45, and 90 min, after which they were stopped with 82.5 µL of 30% trifluoracetic acid. Cleaved Fps were isolated by centrifugation (17000 RPM at 4 °C for 30 min). Samples were run in duplicate.

HPLC analysis was performed on a Prominent (Shimadzu, Prague, Czech Republic) HPLC, with UV detection at 215 nm according to Suttnar et al. [41]. Briefly, 50 µL of the sample was separated on a Jupiter 4µ Proteo 90R, 150 × 2 mm, 4 µm (Phenomenex, Prague, Czech Republic) by the gradient profile t[min]/%B 0/0 24/19 24.1/100 26/100 26.1/0 60/0, and processed with LabSolutions software (Shimadzu, Prague, Czech Republic). Data analysis was done using GraphPad Prism^®^ version 9.1.2 (GraphPad Software, San Diego, CA, USA).

### 4.7. Statistical Analysis

Statistical analyses were carried out using GraphPad Prism^®^ version 9.1.2 (GraphPad Software, San Diego, CA, USA) and MATLAB Online (MathWorks^®^ Inc, Natick, MA, USA). The differences between the control and patients were evaluated by a Kruskal–Wallis test at a significance level of 0.05.

### 4.8. Homology Modeling

Homology modeling with follow-up geometry optimization was performed in Modeller [42], using the crystal structure 3GHG [43] as a template. The crystal structure was shortened to contain amino acids 27—60 of chains A and D (corresponding to fibrinogen chain Aα) (mature protein numbering), 68—92 of chains B and E (=chain Bβ), and 14–35 of chains C and F (=chain γ) (Figure 5). Since a β-hairpin that contains amino acid BβA68 is surrounded by the Aα chain, and these two chains mutually interact (disulfide bridge between BβC65 and Aα’C36), we considered the possible influence of the BβA68S mutation to both Bβ and Aα’ chains. To bring more degrees of freedom into the geometry of the system, we removed specific amino acids in the Bβ and Aα’ chains from the template structure. In total, five differently adjusted template structures were tested, and the best one was chosen according to stereochemical properties and agreement with the crystal structure 3GHG. The best alignment misses amino acids 66–71 of the chain B and 33–37 of the chain D, and the disulfide bridge between amino acids corresponding to BβC65 and Aα’C36 is defined.

To compare structures obtained by the same technique, a model of the above-mentioned fragment of WT fibrinogen was constructed using the same template structure. A set of five models was computed for each model, and the best of them was chosen according to Modeller’s self-evolving function and stereochemical properties determined by Procheck [44]. The RMSDs of structures were determined in SwissPDBViewer [45], and the secondary structure for the whole structures was classified according to Kabsch and Sander [46], as implemented in Procheck. The resulting structures of homology modeling are available in the Supplementary Materials—Coordinates S1 and S2.

## 5. Conclusions

To the best of our knowledge, we have described the four novel fibrinogen variants all located in the *FGB* gene—heterozygous missense BβY416C and BβA68S, homozygous nonsense BβY345*, and heterozygous nonsense BβW403* mutations. We investigated these mutations by a set of biochemical and bioinformatical methods. All patients had decreased levels of functional and plasmatic fibrinogen, except those with BβA68S, which only had lower functional fibrinogen. Functional tests showed abnormal fibrin development in all patients with lower maximal absorbance and impaired fibrin degradation of BβY416C and BβW403* fibrinogens. Moreover, fibrinopeptide cleavage measurements revealed differences between the release of FpB in patients with BβA68S and those of the control. The results obtained from SEM were significantly different in fiber thicknesses for BβY416C, BβA68S, and BβW403* and in fiber densities for BβY416C and BβW403*. Homology modeling showed the impaired protein structure of BβA68S. Collectively, these results indicate that the mutations cause congenital fibrinogen disorders in all patients. Our study demonstrates that the identification, characterization, and modeling of novel fibrinogen variants brings new knowledge to fibrinogen domain structures and interactions that influence the assembly, secretion, and function of this molecule.

## Figures and Tables

**Table 1:** Coagulation screening tests results.

Patient	Fbg Clauss [g/L] (1.9–4.2)	Fbg Total [g/L] (1.8–4.2)	aPTT-INR (0.8–1.2)	PT-INR (0.8–1.2)	TT-INR (0.85–1.15)	RT [s]	Clinical Phenotype	Mutation
P1	1.3	1.7	1.0	1.1	1.2	22.4 (18.1–22.1)	A	BβY416C
P2	1.6	2.9	1.1	1.0	1.2	21.0 (18.9–22.9)	epistaxis	BβA68S
M2	1.8	2.9	1.0	1.0	1.2	20.9 (18.9–22.9)	A	BβA68S
B2	2.1	2.1	1.1	1.0	0.9	19.8 (18.9–22.9)	epistaxis	none
P3	<0.2	0.0	>6	NA	>12	>300 (17.2–21.2)	A	BβY345*
M3	2.0	2.0	1.5	1.1	1.0	17.4 (17.2–21.2)	A	NA
F3	1.8	2.0	1.0	1.1	0.9	16.7 (17.2–21.2)	A	NA
P4	1.6	1.7	NA	NA	1.0	18.6 (18.8–22.8)	pulmonary embolism	BβW403*

Abbreviations: P1: patient 1; P2: patient 2; M2: mother of patient 2; B2: brother of patient 2; P3: patient 3; M3: mother of patient 3; F3: father of patient 3; P4: patient 4; aPTT: activated partial thromboplastin time; INR: international normalized ratio; PT: prothrombin time; TT: thrombin time; RT: reptilase time; A: asymptomatic; *—nonsense mutation; NA: not available.

**Table 2:** Plasma and purified fibrinogen polymerization of each patient and control.

	Control	P1	P2	M2	P4
Plasma (1 g/L)					
lag time [s]	140	340	1940	300	NA
maxOD	10.6	6.4	1.3	10.5	NA
slope (maxOD/s × 10^3^)	12.0	2.8	0.5	12.8	NA
T90% [s]	420	1640	2360	540	NA
Fibrinolysis					
T50% [s]	240	300	360	340	NA
T100% [s]	560	1700	UD	1880	NA
Purified Fbg (1 g/L)					
lag time [s]	270	460	960	1360	240
maxOD	10.1	2.8	3.1	2.2	3.0
slope (maxOD/s × 10^3^)	6.2	1.5	1.3	0.9	1.8
T90% [s]	1000	1240	2280	2300	880
Fibrinolysis					
T50% [s]	160	280	180	180	300
T100% [s]	1380	1480	1420	1160	1780

Abbreviations: lag time: time to 0.5 OD; maxOD: maximum optical density; slope: maxOD divided by time to this value; T90%: time to reach 90% of maxOD; T50%: time to 50% of fibrin degradation; T100%: time to 0.03 OD after fibrin degradation; P1: patient 1; P2: patient 2; M2: mother of patient 2; P4: patient 4; UD: undetectable; NA: not available.

**Table 3:** Results of fiber thicknesses and average no. of fibers per 1 µm^2^ analysis.

Patient	Fiber Thickness [nm]	Average No. of Fibers per 1 µm^2^
control	78.0 ± 22.6	15.0 ± 2.6
P1	109.4 ± 33.8	9.5 ± 2.6
P2	79.6 ± 23.5	16.7 ± 2.6
M2	88.0 ± 27.2	12.1 ± 2.9
P4	118.8 ± 34.5	6.7 ± 2.0
*p*-Value	*p* < 0.0001	*p* < 0.0001

Abbreviations: P1: patient 1; P2: patient 2; M2: mother of patient 2; P4: patient 4.

## Data Availability

The data presented in this study are available on request from the corresponding author.

## Supplementary Materials

The following supporting information can be downloaded at: https://www.mdpi.com/article/10.3390/ijms23020721/s1.
